# Tuberculosis burden in an urban population: a cross sectional tuberculosis survey from Guinea Bissau

**DOI:** 10.1186/1471-2334-10-96

**Published:** 2010-04-16

**Authors:** Morten Bjerregaard-Andersen, Zacarias J da Silva, Pernille Ravn, Morten Ruhwald, Paul L Andersen, Morten Sodemann, Per Gustafson, Peter Aaby, Christian Wejse

**Affiliations:** 1 Bandim Health Project, INDEPTH Network, Apartado 861, 1004 Bissau Codex, Guinea-Bissau; 2 Department of Infectious Diseases, Aarhus University Hospital Skejby, Brendstrupgaardvej, 8200 Aarhus N, Denmark; 3 Department of Infectious Diseases 144, Copenhagen University Hospital Hvidovre, Kettegaards Allé 30, 2650 Hvidovre, Denmark; 4 Department of Epidemiology, Statens Serum Institut, Artillerivej 5, 2300 Copenhagen S, Denmark; 5 Department of Infectious Diseases, Odense University Hospital, Sdr. Boulevard 29, 5000 Odense C, Denmark; 6 Infectious Diseases Research Group, Department of Clinical Sciences, SE-205 02 Malmö, Lund University, Sweden

## Abstract

**Background:**

Little is known about the prevalence of pulmonary tuberculosis (TB) in low income countries. We conducted a cross sectional survey for pulmonary TB and TB symptoms in Bissau, Guinea-Bissau, in an urban cohort with known HIV prevalence. TB surveillance in the area is routinely based on passive case finding.

**Methods:**

Two cohorts were selected based on a previous HIV survey, but only 52.5% of those enrolled in the adult cohort had participated in the HIV survey. One cohort included all adults living in 384 randomly selected houses; in this cohort 8% (135/1687) were HIV infected. The other included individuals 50 years or older from all other houses in the study area; of these 11% (62/571) were HIV infected. Symptom screening was done through household visits using a standardised questionnaire. TB suspects were investigated with sputum smear microscopy and X-ray.

**Results:**

In the adult cohort, we found 4 cases among 2989 individuals screened, giving a total TB prevalence of 134/100,000 (95% CI 36-342/100,000). In the >50 years cohort, we found 4 cases among 571 individuals screened, giving a total prevalence of 701/100,000 (191-1784/100.000). Two of the eight detected TB cases were unknown by the TB program. Of the total TB cases five were HIV uninfected while three had unknown HIV status. The prevalence of TB symptoms was 2.1% (63/2989) and 10.3% (59/571) in the two cohorts respectively.

**Conclusions:**

In conclusion we found a moderately high prevalence of pulmonary TB and TB symptoms in the general population, higher among elderly individuals. By active case finding unknown cases were detected. Better awareness of TB and its symptoms needs to be promoted in low income settings.

## Background

Tuberculosis (TB) is a global emergency; each year 1.7 million people die of TB[1]. In Sub-Saharan Africa the situation is particularly critical, often because HIV fuels the TB epidemic [2,3].

Yet, there are still major gaps in our epidemiological knowledge [4]. In low resource settings the TB incidence rates available are often of poor accuracy and the true spread of the disease is therefore unknown [5]. Good TB prevalence data are even more scarce though some surveys from around the world are available [6-15]. It is possible that the true magnitude of the epidemic is underestimated and many TB cases might never be diagnosed [5]. Left untreated, a TB patient can infect between 10 and 15 persons per year [16]. Such unknown cases, especially the more infectious sputum smear positive [17], could therefore play an important role in the spread of the disease and remain a challenge to TB programs.

We here report the findings of a cross sectional TB survey performed in Bissau, Guinea-Bissau, West Africa. The study was conducted in a cohort where the HIV prevalence was recently assessed making a combined estimate possible. The main objective was to determine the prevalence of active TB and TB symptoms in the overall adult population in a sample of 384 houses as well as in the total population above 50 years of age.

## Methods

### Study population

The study was conducted at the Bandim Health Project (BHP), Bissau, Guinea-Bissau in West Africa, a Demographic Surveillance Site. The BHP is a Danish-Guinean research station, with a study area consisting of 6 suburban districts in the capital Bissau and a total study population of 92,000. All individuals are registered with ID-number, age, sex, ethnic group and socio-economic characteristics. The information is updated through regular censuses.

An extensive TB surveillance system based on passive case finding has been in place since 1996 [18]. A previous study showed that TB is highly endemic in Bissau with an incidence of 471/100,000 for adults [18]. All known TB cases occurring in the study area are registered in a central database at the BHP. The information is obtained at local health centres and the national TB hospital.

Deceased TB patients and selected suspects have a verbal autopsy performed. This consists of a thorough interview of close relatives regarding symptoms and clinical signs preceding death. The questionnaire is afterwards reviewed by two physicians to establish the cause of death and a third physician in case of discrepancy. Although a more imprecise measure, the verbal autopsy method has been validated as a useful tool in developing countries [19].

### Study cohort

Since HIV is one of the most important factors in the global TB epidemic [2,3], the study cohort was based on an HIV survey conducted from May 2004 to January 2007 [20]. The individuals eligible for the HIV survey were divided into two cohorts. One consisted of all adult individuals above 15 years of age living in 384 randomly selected houses in three of the suburban districts (Bandim I, Bandim II and Belem), accounting for ~10% of all houses in that area. This cohort was chosen to provide a community sample of the distribution of HIV-1 and HIV-2 and is denoted the *"adult cohort*". The other cohort included individuals 50 years or older from the rest of the houses in the three suburban districts, i.e. houses not among the 384 random houses. This cohort was selected in order to examine whether elderly people differ in terms of HIV-1 and HIV-2 distribution and is denoted ">*50 years cohort*".

The current TB survey used the same inclusion criteria and was conducted from February 2006 to July 2007. However, a re-census was done in the houses in the adult cohort to account for a high mobility in the study area. This can partly be explained by migration between urban and rural areas [21].

Due to the high mobility, an HIV status was not available for all individuals in the adult cohort. The census prior to the TB survey identified 3714 eligible individuals, while the census prior to the HIV survey had identified a total of 3232 eligible individuals in the adult cohort. The overlap between eligible individuals was 71.9% (2669/3714), i.e. 2669 eligible individuals from the HIV survey were identified again among the 3714 eligible for the TB survey. Of the overlapping 2669 eligible individuals, the HIV survey had obtained an HIV status for 1950 persons. An HIV status was therefore available for 52.5% (1950/3714) of the eligible individuals in the adult cohort in the current TB survey.

In the >50 years cohort individuals were only included if they had been included in the HIV survey. Thus, an HIV status was available for all individuals in this cohort.

### Procedures

The study was based on TB detection by active case finding using house to house visits. Individuals not located at first house visit were attempted located on at least once subsequent visit. The primary screening took place where people lived. Two teams consisting of trained field assistants visited the eligible individuals and screened them for TB symptoms using standardized questionnaires [4]. Individuals were asked for presence of the following TB symptoms: cough, expectorate, haemoptysis (coughing blood), breathlessness, chest pain, fever, nightly sweats, fatigue, weight loss and loss of appetite. Individuals with cough or two other TB symptoms were referred to a physician for further clinical investigation. Sputum smear and X-ray was ordered if the physician also considered the referred individual a TB suspect. If an individual was HIV infected, one TB symptom alone would indicate referral. Individuals reporting haemoptysis were always referred. Referred individuals who did not appear at the health centres were re-invited at least once.

### TB case definitions

The diagnosis of TB was made according to WHO guidelines [17]. TB culture was not available as diagnostic facilities were destroyed during a civil war [22]. Suspects with two or more sputum smears positive at direct microscopy for acid fast bacilli were regarded as smear positive TB cases. Smear negative individuals with clinical symptoms and X-ray signs of pulmonary TB were treated with antibiotics for two weeks and then re-evaluated. If the symptoms persisted and the X-ray was still compatible with TB the person was regarded as a smear negative TB case. Individuals in anti-TB therapy at time of screening and registered through the TB surveillance were defined as "known cases", whereas TB cases not registered were classified as "unknown cases". TB cases were treated according to national guidelines. An HIV treatment program is currently being implemented in Bissau, but was not available during the study. Patient delay for confirmed TB patients was defined as the period between onset of any TB symptom and treatment initiation [23].

### Ethics and consent

Ethical permission was obtained from "Unidade de Coordinação de Estudos e Pesquisas em materio de Saúde", the National Ethical Committee of The Ministry of Health in Guinea-Bissau. Written informed consent, either by signature or fingerprint, was obtained for all screened individuals.

### Statistical analysis

Data was entered using dBASE V software. As age in the two cohorts was not normally distributed, median values were used and age comparisons between groups were performed using the wilcoxon rank-sum test. For dichotomous variables the chi-square test was used. A two-sided P < 0.05 was considered significant. Prevalence of TB symptoms according to HIV status was adjusted to the age distribution in the total study cohort. When calculating an overall estimate of the prevalence of TB symptoms the number of individuals referred to clinical consultation was used. All statistical analysis in this study was done using STATA 9.0 software (Stata Corporation, College Station, Texas, USA).

### Sample size

The previous study found a smear positive TB incidence of 177/100.000 per year [18]. Assuming the TB program only detects about half of the TB cases, we expected a true smear positive TB incidence of 354/100.000. Using a global estimate of duration of disease of approximately one year [5], we would also expect a smear positive TB prevalence of 354/100.000 since Prevalence = Incidence * Duration of disease [5]. Assuming approx. 3000 individuals could be screened in the adult cohort during one year, we would expect a 95% confidence interval of 183-665/100.000, which was considered acceptable for the study.

## Results

The study cohort consisted of 3714 eligible individuals in the adult cohort and 718 in the >50 years cohort. It was possible to locate 2989 (80.5%) and 571 (79.5%) individuals from the two cohorts, respectively. Individuals not located were either at work, at the market, travelling or for some other reason absent. A total of 26 individuals who declined to participate were included among those not screened. Among screened individuals, the median age in the adult cohort was 28.1, 44.0% (1315/2989) were male. In the >50 years cohort the median age was 62.3, 42.2% (241/571) were male. Baseline characteristics are shown in Additional file 1, Table 1. In the adult cohort, screened individuals were younger than those not screened and were more often women. In the >50 years cohort there were no significant differences between the groups.

**Figure 1:**
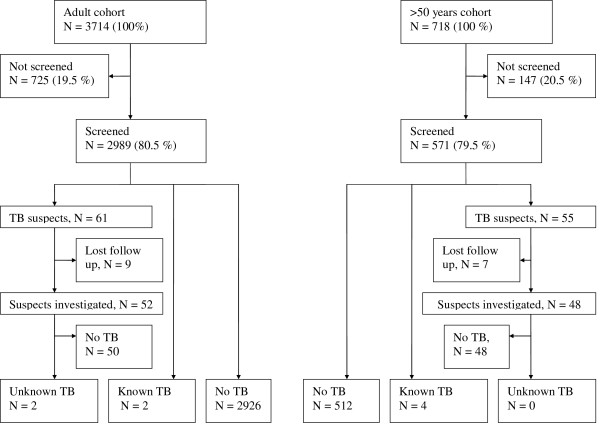
**Study flowchart**. Results of inclusion, screening and TB investigation.

The results of the screening and subsequent TB examinations are displayed in Figure 1. Symptom screening identified 61 suspects of TB in the adult cohort and 55 in the >50 years cohort. Median age among suspects in the adult cohort was 33.8 and 23.0% (14/61) were male. Among suspects from the >50 years cohort the median age was 61.9 and 34.6% (19/55) were male. Total HIV prevalence was 38.5% (15/39) and 20.0% (9/45) in the two groups, respectively.

Of the TB suspects referred, 52 and 48 from the respective cohorts appeared for investigation at the local health centres. Previously unknown TB was diagnosed in two females from the adult cohort aged 24 and 50 year. One was smear positive, the other smear negative. Both were HIV uninfected and reported no contact with other TB patients. One of the two patients left the study area prior to TB treatment initiation. In total 14 TB suspects had X-ray findings which could be suggestive of pulmonary TB but turned out to be sputum smear negative. Smear negative TB was ruled out by secondary X-rays or effect of antibiotic treatment. Differences between X-ray findings in two cohorts were in general not observed although two patients from the >50 years cohort had cicatricial X-ray findings suggestive of former TB. Through the TB registration system 6 known cases were identified among participants from the two cohorts, 2 from the adult cohort and 4 from the >50 years cohort. Two were males. Three were HIV negative, for the three remaining HIV status was not available. Thus, based on 4/2989 TB cases in the adult cohort and 4/571 in the >50 years cohort we calculated a total prevalence of 134/100,000 and 701/100,000, respectively. TB prevalence estimates are displayed in Additional file 1, Table 1.

One TB suspect in the >50 years cohort died before a diagnosis was established. A verbal autopsy determined that TB was the most likely cause of death, but this person has not been included as a TB case as a verbal autopsy diagnosis has a greater degree of uncertainty. If the person had been included, the TB prevalence in the >50 years cohort would have been 876/100,000 (285-2032/100,000).

Among the 6 known TB cases found in the two cohorts, clinical data was available for 3 patients in whom treatment delay was 1, 2 and 4 months. The mean delay (± SD) was 70 days (± 46). For the two unknown TB cases found the delay was two weeks and 6 months, respectively.

Apart from self-reported weight loss, TB symptoms in general were not common in the two cohorts. The overall prevalence of TB symptoms in the adult cohort was 2.1% (63/2989) and 10.3% (59/571) in the >50 years cohort, both estimates including individuals with known active TB. Additional file 2, Table 2 shows age-adjusted prevalence estimates of TB symptoms in the adult cohort, irrespective of duration, and stratified by HIV status.

## Discussion

Our study provides population-based data on the prevalence of active TB and TB symptoms in a low resource setting with a well described HIV burden. The main finding was a moderate burden of unknown TB and TB symptoms in an area with ongoing TB surveillance based on passive case finding. The number of unknown smear positive TB was 1 out of a total of 5 smear positive cases. The total TB prevalence in the community was in the order of 134/100,000, but much higher among elderly individuals.

Only few other TB prevalence studies have been conducted in Sub-Saharan Africa and only one with a combined HIV estimate. A survey from Ethiopia identified a smear positive TB prevalence of 78/100,000 by using symptom screening [11]. The burden of previously unknown smear positive TB was higher in the Ethiopian study, since there was one unknown smear positive case for every two cases known by the TB program. By using fluorescence microscopy as screening method, a study from Eritrea identified a total smear positive prevalence of 90/100,000 in the adult population [10]. A recent survey from South Africa encountered a much higher burden of previously undiagnosed TB, i.e. 1.575/100,000 out of a total prevalence of 2.517/100,000 [14]. The high TB prevalence in South Africa was mainly attributed to an HIV-1 prevalence of around 23%.

The previous survey in our study area found a smear positive TB incidence of 177/100,000 [18]. Defining duration of disease as the 8 months of TB treatment, one would expect a prevalence of known smear positive TB of 177/100,000 * 8/12 = 118/100,000, which is in the same order as the prevalence encountered in the present study.

Our data also suggests a long delay between onset of symptoms and treatment initiation (approx. 2 months). The reason for this remains unknown, but a lack of TB awareness and reliance on private healers could play a role [23,24]. Also, repeated visits to health clinics without correct diagnosis could be an explanatory factor [23]. Finally, it is worth noting that the two unknown cases were women. Female sex has previously been associated with undiagnosed TB [11] and a longer patient delay [23].

TB symptoms were only moderately prevalent in the adult cohort, but the prevalence was higher among elderly. The Ethiopian study found 2.6% of the adult population to be symptomatic [11], which is close to our findings. In the adult cohort most TB symptoms were more prevalent among HIV-2 infected, but this also reflects a higher morbidity among elderly, who are predominately HIV-2 infected. Thus, no correlation between HIV status and TB symptoms was established after age-adjustment. The fact that the prevalence symptoms among HIV-1 and HIV uninfected was in the same order most likely reflects that the HIV-1 epidemic in Bissau is still relatively young [20] and most HIV-1 infected remain asymptomatic.

Since TB surveys are costly [25], the most effective way of targeting hidden TB and reducing patient delay is probably increased education of health care providers and TB information campaigns. The focus should be symptoms, modes of transmission and that it is most likely a curable condition.

Our TB survey has several limitations. Since the TB survey was performed in a cohort with a well described HIV burden the sample size is relatively small and estimates are therefore subject to a considerable uncertainty error. Also, some individuals could have been HIV infected between the HIV and the TB survey, thereby biasing some of the results. Another limitation is that the overlap between the HIV and TB survey was low, only 52.5% of screened individuals in the adult cohort had a known HIV status. This reflects that the study population is highly mobile. We could have chosen to include only individuals with known HIV status, but preferred to include all adults in order to obtain the most recent and precise community sample and to avoid introducing the bias of enrolling only the non-mobile individuals known from the HIV survey for TB screening.

As Bissau is a resource limited setting we chose a symptom screening model. Although logistically feasible this increases the risk of overlooking TB patients that were asymptomatic or under-reporting symptoms [14]. The examination of symptomatic patients could have been further improved by using sputum culture, as this offers the highest sensitivity in both HIV infected and uninfected patients [26]. Unfortunately this was not possible at the time of study as diagnostic facilities were destroyed during a civil war [22]. TB culture is currently being implemented in Bissau.

Ideally, in a TB prevalence survey sputum smear and culture should be performed on all screened individuals. This is especially true in areas where TB-HIV co-infection is the major factor, since culture here offers a markedly higher sensitivity [26]. However, any TB survey must take logistical and financial constraints into consideration but also outweigh the costs and benefits of improved screening vs. expanding the sample size [4]. Since most individuals screened reported no symptoms, a future survey in our study area could include an interview with the heads of the families in each household followed by symptom screening and examination only of those reported symptomatic. Although a number of TB cases would probably be missed and no HIV status would be available, a much larger area could be covered thereby increasing the sample size and potentially diagnosing many unknown TB cases.

## Conclusions

We found a moderately high burden of pulmonary TB and TB symptoms in this urban setting, especially among elderly. Unknown cases were detected and we further noted a long patient delay prior to treatment initiation. The study underscores the need to conduct TB awareness campaigns among both health personnel and the general population in high endemic settings.

## Competing interests

The authors declare that they have no competing interests.

## Authors' contributions

MB-A, CW, PAA and PLA designed the study. MB-A, ZJdS and PAA supervised the inclusion and clinical examinations in Guinea Bissau. PR, MR, MS and PG co-designed the study and assisted with questions throughout the process. All authors approved of the final version of the manuscript.

## Pre-publication history

The pre-publication history for this paper can be accessed here:

http://www.biomedcentral.com/1471-2334/10/96/prepub

## Supplementary Material

Additional file 1 **Table 1**. Characteristics and findings of study sample for the two cohorts. Click here for file

Additional file 2 **Table 2**. Sex, age and TB symptoms stratified by HIV status in the adult cohort. Click here for file
