# Supplementary material for: Tuberculosis burden in an urban population: a cross sectional tuberculosis survey from Guinea Bissau
Source: BMC Infect Dis. 2010 Apr 16;10:96. doi: 10.1186/1471-2334-10-96 (PMC2860354; doi:10.1186/1471-2334-10-96)
Supplement: Additional file 1 — Table 1. Characteristics and findings of study sample for the two cohorts. [file 1471-2334-10-96-S1.DOC]

**Table 1: Characteristics and findings of study sample for the two cohorts**

|  | ***Adult cohort***  *Screened*  *N= 2989* | ***Adult cohort***  *Not Screened*  *N= 725* | ***P*** | ***>50 years cohort***  *Screened*  *N= 571* | ***>50 years cohort***  *Not screened*  *N= 147* | ***P*** |
| --- | --- | --- | --- | --- | --- | --- |
| ***Demographics*** |  |  |  |  |  |  |
| Male sex | 1315 (44.0) | 385 (53.1) | <0.001 | 241 (42.2) | 59 (40.1) | 0.65 |
| Median age | 28.1 | 32.1 | <0.001 | 62.3 | 64.1 | 0.26 |
| HIV-1 prevalence | 63/1687 (3.7) | 14/263 (5.3) | 0.22 | 2/571 (0.4) | 0/147 (0) | 0.47 |
| HIV-2 prevalence | 64/1687 (3.8) | 13/263 (4.9) | 0.37 | 59/571 (10.3) | 16/147 (10.9) | 0.85 |
| HIV-1+HIV-2 prevalence | 8/1687 (0.5) | 4/263 (1.5) | 0.04 | 1/571 (0.2) | 0/147 (0) | 0.61 |
| ***TB cases*** |  |  |  |  |  |  |
| Total TB cases | 4 | --- | --- | 4 | --- | --- |
| Unknown TB cases | 2 | --- | --- | 0 | --- | --- |
| Total smear positive TB cases | 3 | --- | --- | 2 | --- | --- |
| Unknown smear positive TB cases | 1 | --- | --- | 0 | --- | --- |
| ***TB prevalence*** |  |  |  |  |  |  |
| Total TB prevalence | 134/100.000  (36-342/100.000) | --- | --- | 701/100.000  (191-1784/100.000) | --- | --- |
| Unknown TB prevalence | 67/100.000  (8-242/100.000) | --- | --- | 0 | --- | --- |
| Total smear positive TB prevalence | 100/100.000  (21-293/100.000) | --- | --- | 350/100.000  (43-126/100.000) | --- | --- |
| Unknown smear positive TB prevalence | 33/100.000  (8-186/100.000) | --- | --- | 0 | --- | --- |

Cells are N (%). P-values are for comparing screened and not screened individuals. HIV status was available for 1950 in the adult cohort and 718 in the >50 years cohort. 95% CI is included in TB prevalence results.
