# Supplementary material for: Tuberculosis burden in an urban population: a cross sectional tuberculosis survey from Guinea Bissau
Source: BMC Infect Dis. 2010 Apr 16;10:96. doi: 10.1186/1471-2334-10-96 (PMC2860354; doi:10.1186/1471-2334-10-96)
Supplement: Additional file 2 — Table 2. Sex, age and TB symptoms stratified by HIV status in the adult cohort. [file 1471-2334-10-96-S2.DOC]

Table 2: Sex, age and TB symptoms stratified by HIV status in the adult cohort.

|  | ***HIV uninfected***  *N= 1552* | | ***HIV-1 infected***  *N= 63* | | ***HIV-2 infected***  *N= 64* | | ***HIV-1+HIV-2 infected***  *N= 8* | |
| --- | --- | --- | --- | --- | --- | --- | --- | --- |
| Male sex | 604 (38.9) | | 25 (39.7) | | 20 (31.3) | | 1 (12.5) | |
| Mean age | 32.9 | | 35.2 | | 48.8 | | 42.3 | |
|  | *Crude* | *Adjusted** | *Crude* | *Adjusted** | *Crude* | *Adjusted** | *Crude* | *Adjusted** |
| Cough | 20 (1.3) | 20.4 (1.3) | 1 (1.6) | 0.6 (0.9) | 4 (6.3) | 1.1 (1.7) | 1 (12.5) | 0.1 (1.2) |
| Expectorate | 18 (1.2) | 18.5 (1.2) | 1 (1.6) | 0.6 (0.9) | 2 (3.1) | 0.3 (0.4) | 1 (12.5) | 0.1 (1.2) |
| Breathlessness | 23 (1.5) | 22.8 (1.5) | 1 (1.6) | 0.9 (1.4) | 6 (9.4) | 1.5 (2.3) | 1 (12.5) | 0.1 (1.2) |
| Chest pain | 28 (1.8) | 27.1 (1.8) | 1 (1.6) | 0.8 (1.3) | 3 (4.7) | 0.9 (1.4) | 0 (0) | 0 (0) |
| Fever | 73 (4.7) | 73.4 (4.7) | 2 (3.2) | 2.3 (3.7) | 7 (10.9) | 2.0 (3.1) | 1 (12.5) | 0.2 (2.4) |
| Night sweats | 6 (0.4) | 5.7 (0.4) | 0 (0) | 0 (0) | 2 (3.1) | 0.6 (1.0) | 0 (0) | 0 (0) |
| Fatigue | 13 (0.8) | 12.9 (0.8) | 1 (1.6) | 0.8 (1.3) | 2 (3.1) | 0.3 (0.5) | 0 (0) | 0 (0) |
| Weight loss | 156 (10.0) | 160.2 (10.3) | 10 (15.9) | 6.3 (10.0) | 16 (25.0) | 3.3 (5.2) | 3 (37.5) | 0.4 (5.2) |
| Loss of appetite | 19 (1.2) | 19.0 (1.2) | 3 (4.8) | 1.8 (2.9) | 3 (4.7) | 0.6 (1.0) | 1 (12.5) | 0.2 (2.4) |

Cells are N (%). The table includes individuals with active TB.

*Adjusted for age
